# ROS and Sympathetically Mediated Mitochondria Activation in Brown Adipose Tissue Contribute to Methamphetamine-Induced Hyperthermia

**DOI:** 10.3389/fendo.2013.00044

**Published:** 2013-04-23

**Authors:** Manuel Sanchez-Alavez, Bruno Conti, Malcolm R. Wood, Nikki Bortell, Eduardo Bustamante, Enrique Saez, Howard S. Fox, Maria Cecilia Garibaldi Marcondes

**Affiliations:** ^1^Chemical Physiology Department, The Scripps Research InstituteLa Jolla, CA, USA; ^2^Core Microscopy Facility, The Scripps Research InstituteLa Jolla, CA, USA; ^3^Molecular and Cellular Neuroscience Department, The Scripps Research InstituteLa Jolla, CA, USA; ^4^Department of Pharmacology and Experimental Neurosciences, University of Nebraska Medical CenterOmaha, NE, USA

**Keywords:** methamphetamine, hyperthermia, brown adipose tissue, mitochondria, thermogenesis

## Abstract

Methamphetamine (Meth) abuse has been shown to induce alterations in mitochondrial function in the brain as well as to induce hyperthermia, which contributes to neurotoxicity and Meth-associated mortality. Brown adipose tissue (BAT), a thermogenic site known to be important in neonates, has recently regained importance since being identified in significant amounts and in correlation with metabolic balance in human adults. Given the high mitochondrial content of BAT and its role in thermogenesis, we aimed to investigate whether BAT plays any role in the development of Meth-induced hyperthermia. By ablating or denervating BAT, we identified a partial contribution of this organ to Meth-induced hyperthermia. BAT ablation decreased temperature by 0.5°C and reduced the length of hyperthermia by 1 h, compared to sham-operated controls. BAT denervation also affected the development of hyperthermia in correlation with decreased the expression of electron transport chain molecules, and increase on PCG1a levels, but without affecting Meth-induced uncoupling protein 1 upregulation. Furthermore, in isolated BAT cells in culture, Meth, but not Norepinephrine, induced H_2_O_2_ upregulation. In addition, we found that *in vivo* Reactive Oxygen Species (ROS) play a role in Meth hyperthermia. Thus, sympathetically mediated mitochondrial activation in the BAT and Meth-induced ROS are key components to the development of hyperthermia in Meth abuse.

## Introduction

d-Methamphetamine HCl Methamphetamine (Meth) is a powerful stimulant with potent addictive and neurotoxic properties. It is illegally used for various reasons, including weight loss, alertness, and enhancement of sexual pleasure, and is popular due to its price, availability, and effects. Meth abuse can lead to CNS effects that include depression and psychosis (Winslow et al., 2007), in addition to exposure to HIV (Koopman et al., 1994; Rotheram-Borus et al., 1994a,b; Chesney et al., 1998; Molitor et al., 1998; LaVoie et al., 2004) and other infections (van Griensven et al., 2001; Beyrer et al., 2004; Lee et al., 2005; Patterson et al., 2006; Vogt et al., 2006; Cohen et al., 2007; Kaye et al., 2007; Sutcliffe et al., 2009). However, uncontrolled hyperthermia is a major cause of mortality in Meth abuse (Gowing et al., 2002). Therefore, the recognition and management of hyperthermic reactions are essential for saving lives.

In the brain, Meth is a substrate for the Dopamine (DA) transporter, which allows the drug access to DAergic neurons, where it inhibits the vesicular monoamine transporter, increasing DA in the synaptic cleft. This can result in nerve terminal damage and neurotoxicity through mechanisms that are not yet fully understood. However, monoamine metabolites, excitotoxic neurotoxicity, free radicals, and metabolic stress are potential candidates (Rippeth et al., 2004). Hyperthermia accentuates excitotoxic neurotransmitter release, increases oxygen free radicals, accelerates cytoskeletal protein degradation, and increases the risk of seizures (Baena et al., 1997; Ginsberg and Busto, 1998). Furthermore, oxidative stress in correlation with astrocytosis and inflammatory molecules in the brain seem to play an important role in the neurotoxicity associated with Meth hyperthermia (Cadet et al., 1994; Sheng et al., 1994; Fukumura et al., 1998a,b). How the mechanisms by which Reactive Oxygen species (ROS) are generated in Meth abuse, in addition to how ROS generated in peripheral sites such as brown adipose tissue (BAT) contribute to the hyperthermia phenomenon, have not been extensively explored.

Hyperthermia may result from a combination of several components. One may be UCP3, which contributes to the generation of heat in the muscle (Mills et al., 2003; Sprague et al., 2007). Another may be BAT. Recent studies using PET scans have shown that BAT is still present in human adults dorsally, in the upper chest and neck (Nedergaard et al., 2007), along the spinal cord, in the mediastinum (Truong et al., 2004), and intra-diaphragmatically (Bar-Shalom et al., 2004). Therefore, the participation of BAT in adult thermogenic processes may be underestimated and it should henceforth be considered an organ of physiological importance. These BAT deposits in adults become more metabolically active upon cold exposure and less active upon the administration of adrenergic β-blockers (Nedergaard et al., 2007). Thus, the activation of BAT is important for energy dissipation. It has been reported that outflow pathways from the CNS may mediate differential inhibitory control of sympatho-excitatory BAT thermogenesis (Nakamura and Morrison, 2008). However, the role of BAT in Meth-induced hyperthermia has not been previously explored. Here we have designed experiments to identify morphological and biochemical changes in BAT following Meth-induced hyperthermia in mice. We have identified a role for sympathetic input in the induction of excitatory pathways in BAT mitochondria, and a role for ROS *in vivo*, in the development of hyperthermia following Meth injection.

## Materials and Methods

### Mice and telemetry

All procedures were approved by the Institutional Animal Care and Use Committee of the Scripps Research Institute and were carried out on male C57BL/6J mice (20–25 g/3 months old) and maintained on regular chow diet {Harlan Teklad LM-485 Diet 7012 (65% carbohydrate (kcal), 13% fat, metabolizable energy 3.41 kcal/g)}. Access to food and water were *ad libitum*. For telemetry studies, mice were anesthetized with isoflurane (induction 3–5%, maintenance 1–1.5%) and surgically implanted with radio transmitter telemetry devices (TA-F20, Data Sciences, Inc.) into the peritoneal cavity for core body temperature (CBT) and motor activity (MA) measurements. Mice were allowed to recover for 2 weeks and then submitted for freely moving telemetry recording (each group *n* = 4–6) simultaneously with the CLAM system (see below). Mice were individually housed in clear respiratory chambers in a room maintained at 25 ± 0.5°C on a 12:12 h light–dark cycle (lights on at 6:00 a.m.). The respiratory chambers were positioned onto receiver plates (RPC-1; Data Sciences) and radio signals from the implanted transmitter were continuously monitored and recorded. The animal’s CBT and MA (number of horizontal movements) were monitored with a fully automated data acquisition system (Dataquest A.R.T., Data Sciences, Inc.) for at least 72 h before Meth and other treatments, to ascertain that baseline levels of temperature were stable and that no ongoing febrile response confounded results.

### Respiratory exchange ratio

Indirect calorimetry was performed simultaneously with telemetry in 3–4 days-acclimated, singly housed, standard diet-fed mice using a computer-controlled, open-circuit system (Oxymax System) that is part of an integrated Comprehensive Lab Animal Monitoring System (CLAMS; Columbus Instruments, Columbus, OH, USA). Animals were tested in clear respiratory chambers (20 cm × 10 cm × 12.5 cm) with a stainless steel elevated wire floor. Each of these chambers was equipped with a food tray connected to a balance. Room air was passed through chambers at a flow rate of ∼0.5 l/min. Exhaust air from each chamber is sampled at 30-min intervals for 1 min. Sample air was sequentially passed through O_2_ and CO_2_ sensors (Columbus Instruments) for determination of O_2_ and CO_2_ content, from which measures of oxygen consumption (VO_2_) and carbon dioxide production (VCO_2_) were estimated. Outdoor air reference values were sampled after every four measurements. Gas sensors were calibrated prior to the onset of experiments with primary gas standards containing known concentrations of O_2_, CO_2_, and N_2_ (Airgas Puritan Medical, Ontario, CA, USA). Respiratory exchange ratio (RER) was calculated as the ratio of VCO_2_/VO_2_. The caloric value (CV) was calculated using the following constant: (3.815 + 1.232 × RER). This was used to calculate Heat (kcal/h), as CV × VO_2_ in liters/unit time. VCO_2_ and VO_2_ were normalized with respect to individual animals’ body weight and corrected to an effective mass value. Mice undergoing indirect calorimetry were also acclimated to the respiratory chambers for 3–4 days before the onset of study. Data were recorded under ambient room temperature clamped at 25°C, beginning from the onset of the light cycle 24 h, for 3 days.

### *In vivo* administration of Meth

Mice were exposed to an acute injection of d-Methamphetamine HCl (Alltech Associates Inc., Deerfield, IL, USA; 3.0 mg/kg ip, single dose) 4 h after the lights were on. The animals were sacrificed 24 h after treatment.

### Brown adipose tissue ablation and denervation

Animals were anesthetized with isoflurane (induction 3–5%, maintenance 1–1.5%) and maintained on a feedback regulated warming pad to prevent possible hypothermia, and an incision (approximately 2 cm) was made between the scapulae to expose the interscapular BAT. Mice were maintained on a heat pad until recovery from the anesthesia. In ablation procedures, the whole tissue was isolated from blood supply and innervations and explanted from the animals. Denervation procedures: using a stereomicroscope, intrascapular BAT was carefully moved outward from the surrounding muscle to expose the five intercostal nerve bundles entering each pad and cutting with scissors (Scarpace and Matheny, 1998; Scarpace et al., 1998; Pulinilkunnil et al., 2011) without disrupting the blood supply. Mock surgeries were performed in control mice. The incision was closed with 5–0 vincryl sutures and the animals were allowed to recover for 2 weeks prior to experiments. The viability of denervated BAT was checked histologically upon sacrifice of the animals.

### qRT-PCR and detection of mitochondrial molecules

Total RNA was purified from samples using Totally RNA kit (Ambion, Austin, TX, USA) following the protocol of the manufacturer, with an additional centrifugation step to remove cellular debris. RNA was further purified (RNeasy mini kit; Qiagen, Valencia, CA, USA). CDNA was obtained using RT2 First strand kits (SABiosciences, Qiagen, Frederick, MD, USA) following manufacturer’s instructions. The primer and probe sequences selected for use were either designed for mouse sequences either by reference to previous studies, by the Primer Express software (Applied Biosystems, Foster City, CA, USA), or through the Genescript online tool (https://www.genscript.com/ssl-bin/app/primer). PCR array technology (SABiosciences) was also applied to measure various molecules within proton-transport chain complexes (PAMM-008). The molecules investigated were calculated into relative amounts of mRNA in the samples, by subtracting the average cycle threshold (Ct) of the primary signal for GAPDH from that for each molecule of interest to give changes in Ct (dCt), and the degree of changes in expression (the differences in dCt, or ddCt) was determined by using log2 relative units. Calculations were performed using PCR Array Data Analysis Software (SABiosciences).

### Transmission electron microscopy

Three mice in each group were perfused with 0.9% saline, followed by 4% paraformaldehyde-1.5% glutaraldehyde in 0.1 M cacodylate buffer with 1 mM CaCl_2_. The BAT was removed and immersed in the above fixative on ice for 6 h, and was transferred to 2.5% glutaraldehyde in 0.1 M cacodylate buffer with 1 mM CaCl_2_ for overnight fixation. After a buffer wash, the tissue was further fixed in 1% OsO_4_ with 1.5% potassium ferricyanide in 0.1 M sodium cacodylate and again washed in cacodylate buffer, dehydrated in graded ethanol series, and transitioned in propylene oxide. BAT was embedded in Embed 812/Araldite (Electron Microscopy Sciences, Hatfield, PA, USA). Six thick sections (1–2 μm) were cut from different areas of BAT, mounted on glass slides, and stained in toluidine blue for general assessment with the light microscope. Subsequently, 70-nm thin sections were cut, mounted on copper slot grids coated with Parlodion, and stained with uranyl acetate and lead citrate for examination on a Philips CM100 electron microscope (FEI, Hillsborough, OR, USA) at 80 kV, and images were collected using a Megaview III charge-couple-device (CCD) camera (Olympus Soft Imaging Solutions, Lakewood, CO, USA). The diameter of mitochondria in brown adipocytes was measured in an average of 23 EM coded pictures taken from at 3400× magnification, using Image J 1.43u software (NIH, USA). For morphometric analysis of cristae, mitochondria were randomly selected from coded samples and the number of cristae was counted and normalized for the surface of the organelle calculated by fitting using the Multi Measure plug-in of Image J 1.43u software (NIH).

### BAT cell isolation and measurement of hydrogen peroxide production

Mature brown adipocytes were isolated from adult 6 weeks old C57Bl/6 mice using Collagenase A digestion as described (Klein et al., 1999). Isolated adipocytes were counted, plated in 96 well plates at 2 × 10^5^ cells/well and immediately stimulated with 60 μM Meth, or 500 pg–100 ng/ml Norepinephrine (NE; Sigma Aldrich), or 5 μM of *N*-acetyl-d-cysteine (NAC), or combinations of these reagents. H_2_O_2_ production was measured by the Amplex Red assay (Zhou et al., 1997) (Molecular Probes, Eugene, OR, USA), following manufacturer’s instructions. Fluorescence was measured for 120 min at 37°C, at an excitation wavelength of 545 nm and emission wavelength of 590 nm using a Tecan Infinite F500 apparatus (Tecan Systems, Inc., San Jose, CA, USA). Results from quadruplicates correspond to 60-min reading, and were normalized to a standard curve.

### *In vivo* treatment with *N*-acetyl-d-cysteine

*N*-acetyl-d-cysteine (Sigma Aldrich) was administered intraperitoneally 60 min prior to the injection of Meth or Vehicle at a dose of 1000 mg/kg as a 20% solution described previously (Blackwell et al., 1996).

### Statistical analysis

One-way ANOVA, followed by multiple comparisons Bonferroni’s test were performed using Prism Software (Graphpad software, San Diego, CA, USA). Comparisons in qRT-PCR measurements were performed using PCR Array Data Analysis Software (SABiosciences).

## Results

**Figure 1:**
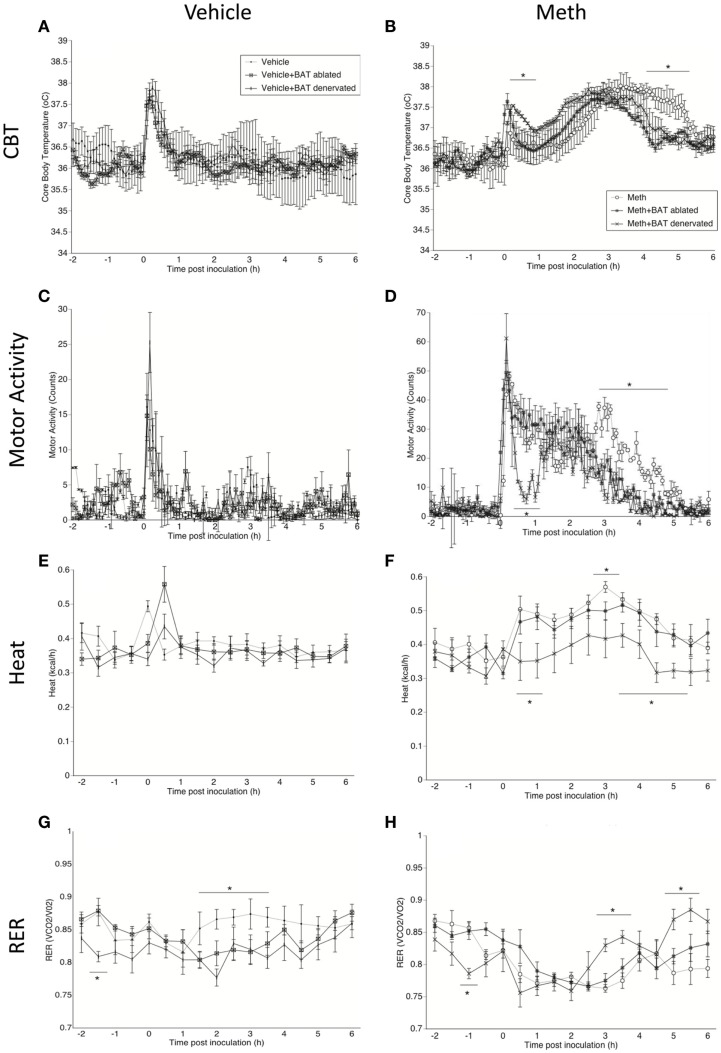
**Characterization of Meth hyperthermia in mice following a single injection of Meth and the contribution of BAT**. Mice implanted with telemetry devices were treated with a single IP injection of Vehicle **(A,C,E,G)** or Meth (3 mg/kg) **(B,D,F,H)**, and were evaluated during 6 h for core body temperature **(A,B)**, motor activity **(C,D)**, heat production **(E,F)**, and RER **(G,H)**. Values represent the average ± SEM of six animals/group. Animals that had BAT surgically ablated or denervated were compared to sham-operated mice. *p < 0.05, *n* = 6, Student *t*-test indicates (hyperthermia).

In order to check if the injection of Meth in mice was able to mimic the hyperthermic effects of the drug in humans, we examined the ability of peripherally injected Meth to cause hyperthermia in C57Bl/6 male mice implanted with telemetry devices (Figure 1). The animals were recorded for baseline CBT and MA prior to and up to 6 h after a single dose of Saline as vehicle or Meth (3 mg/kg). The RER was calculated from ventilated CO_2_ (VCO_2_) divided by ventilated Oxygen (VO_2_) during the same period, and Heat production was derived from VO_2_ measurements normalized with body mass, as described in Section “Materials and Methods.” The influence of BAT was estimated by performing injections in animals that had had the interscapular tissue surgically ablated (BATAbl) in comparison to sham-operated mice. To investigate the role of β-adrenergic input, interscapular BAT was surgically denervated (BATDen).

All animals showed a transient peak in temperature and MA immediately after the injection, which was caused by the stress of manipulation, but there was no significant difference between groups. After the stress peak subsided, a significant increase in CBT starting 1 h after injection, was observed in Meth-injected animals, but not in the vehicle group. In sham-operated Meth-injected animals, CBT increased gradually from 1.5 h post inoculation, reaching a 38°C peak that lasted up to 6 h post inoculation (Figure 1B). BAT ablation reduced both the intensity of hyperthermia, by 0.5°C, and the length of the temperature response, by 1 h (Figure 1B). BAT denervation also affected the CBT profile by reducing its length (Figure 1B).

The increase in temperature caused by Meth correlated with an increase in MA (Figure 1D). Curiously, changes observed both in BAT ablation, and even more drastically, in denervation, correlated with a reduction of MA induced by Meth (Figure 1D). This may suggest that changes in BAT after Meth can affect skeletal muscle activity. Meth alone also induced an increase in heat production, which was correlated to the hyperthermia and MA peaks (Figure 1F). Interestingly, BAT ablation did not affect heat production after Meth, but BAT denervation significantly reduced heat production induced by Meth (Figure 1F). Meth-induced a RER reduction compared to vehicle, and BAT ablation or denervation did not affect the RER profile induced by Meth (Figure 1H). Overall, in vehicle-injected animals, BAT denervation and ablation did not *per se* alter CBT (Figure 1A), MA (Figure 1C), or Heat (Figure 1E). However, both ablation and denervation of BAT caused a transient stress-induced reduction of RER following vehicle injection (Figure 1G), suggesting a shift to fatty acids metabolic utilization.

**Figure 2:**
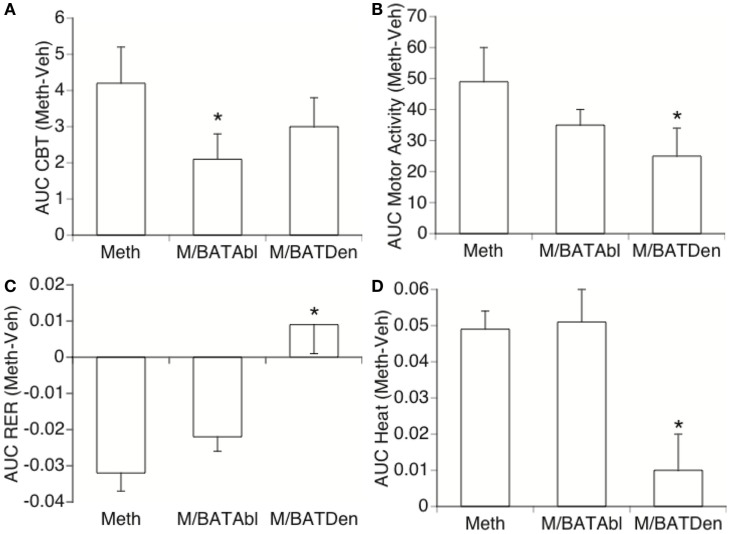
**Effect of BAT on Meth-induced metabolic changes**. Area under the curve (AUC) was used to quantify changes in **(A)** CBT, **(B)** locomotor activity, **(C)** RER, and **(D)** heat production in Meth compared to vehicle-injected animals, and to investigate the effect of BAT ablation or denervation. Stress peak (first hour) was excluded. Values represent the average difference between Meth-injected and Vehicle-injected respective controls ± SEM, for six animals/group. *p < 0.05, ANOVA followed by Bonferroni’s *post hoc* test, *n* = 6 animals/group.

**Figure 3:**
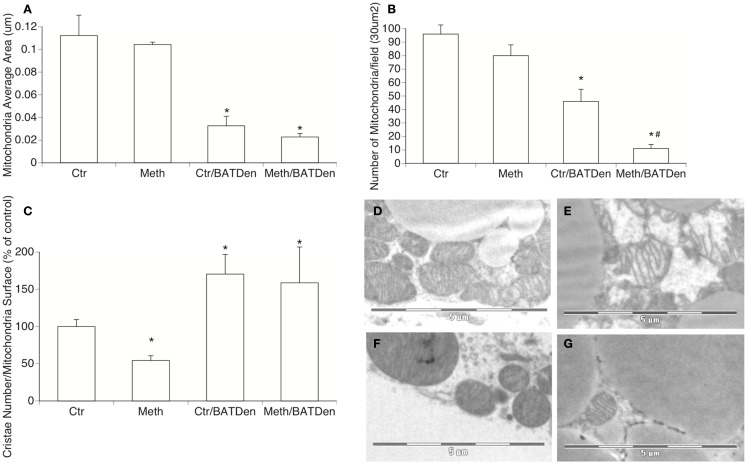
**Mitochondria density and morphology**. Electron microscopy was utilized to investigate changes in density and shape of mitochondria in BAT due to Meth, under sympathetic regulation. The evaluation of mitochondria area **(A)**, number **(B)**, and cristae density **(C)** were performed on captures of BAT tissue at 3400× magnification, using Image J 1.43u software. Representative picture of **(D)** mitochondria from control sham-operated animals, **(E)** mitochondria from Meth-injected BAT, **(F)** mitochondria from control denervated BAT, and **(G)** Meth-injected denervated BAT. *p < 0.05 Compared to control sham-operated group, ^#^*p* < 0.05 compared to control BAT-denervated group. ANOVA followed by Bonferroni’s *post hoc* test, *n* = 3 mice/group.

The analysis of the area under the curve (AUC – Figure 2) was used to quantify the changes caused by Meth and the influence of BAT in the parameters expressed in Figure 1. In order to eliminate the noise of the injection-related stress peak, we calculated the AUC between 1 and 6 h after vehicle or Meth. The AUC applied to CBT (Figure 2A) confirmed that BAT ablation significantly decreased CBT following Meth, and revealed that BAT contributes to 40% of Meth-induced hyperthermia. BAT denervation substantially, but not significantly, affected CBT by 28.5% (Figure 2A). BAT also interfered with MA, with ablation being responsible for a substantial, but not significant, 28.7% decrease, and denervation being responsible for a significant 49% decrease of movement (Figure 2B). The effect of BAT ablation and denervation differed over RER, where denervation, but not ablation, drastically affected RER by preventing 75% of its reduction (Figure 2C). A similar effect was observed regarding heat production, where BAT denervation, but not ablation, significantly prevented the increase of heat by 95% (Figure 3D).

It has been shown that BAT produces heat by uncoupling electron transport from ATP synthesis through a brown fat-specific UCP, uncoupling protein 1 (UCP1), which is present in mitochondria inner membranes (Cannon and Nedergaard, 2004). Therefore, we investigated whether Meth induced changes in BAT mitochondria, and whether these changes were caused by direct actions of the drug, or mediated by Meth-induced NE accessing BAT through sympathetic innervation. Morphologically, Meth caused a reduction in mitochondria cristae density (Figure 3C), without significantly changing the average size or number of mitochondria available in the tissue (Figures 3A,B,E). BAT denervation, regardless of Meth, caused a reduction of the size of individual mitochondria (Figures 3A,F,G), significantly reduced the number of mitochondria in the tissue (Figure 3B), and also increased cristae density (Figure 3C). Meth-injected in BAT-denervated mice caused an even more drastic reduction in the number of mitochondria in the BAT tissue (Figure 3B). This suggests that the metabolic changes observed in Meth-injected BAT ablated animals, and which is at least partially replicated by denervation, can be due to BAT mitochondria activity.

**Figure 4:**
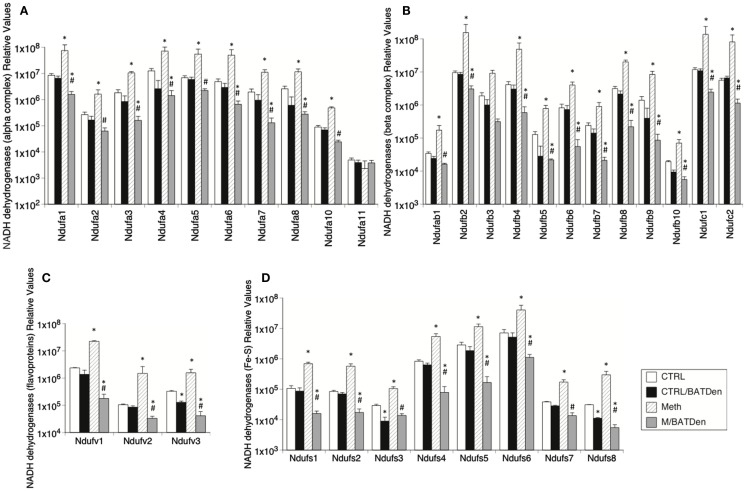
**Effect of Meth on the expression of genes from mitochondrial Complex I**. Transcriptional levels of individual proteins in the Complex I were measured by SyBrGreen qRT-PCR. Relative levels were normalized against the expression of GAPDH. Levels of **(A)** alpha complex NADH dehydrogenases, **(B)** beta complex NADH dehydrogenases, **(C)** Flavoprotein-NADH dehydrogenases, and **(D)** Fe-S NADH dehydrogenases. Values represent the average ± SEM of six animals/group. *p < 0.05 Compared to respective control, ^#^*p* < 0.05 compared to Meth group.

We further investigated the status of mitochondria metabolism by measuring the transcription of mitochondria electron transport chain complex enzymes. The expression of Complex I enzymes was upregulated by Meth and inhibited by denervation (Figure 4). The Complex I NADH alpha and beta dehydrogenases (Figures 4A,B, respectively) were on average, were upregulated 10-fold by Meth (black columns), and on average downregulated fivefold in BAT-denervated, Meth-injected tissue (striped columns), with an ANOVA *p* value = 0.0034 for alpha complex (Figure 4A) and *p* < 0.001 for beta complex (Figure 4B). A similar trend was observed in the expression of flavoproteins (Figure 4C, ANOVA *p* value < 0.001), and in the expression of NADH dehydrogenase Fe-S gene proteins (Figure 4D, ANOVA *p* < 0.001).

**Figure 5:**
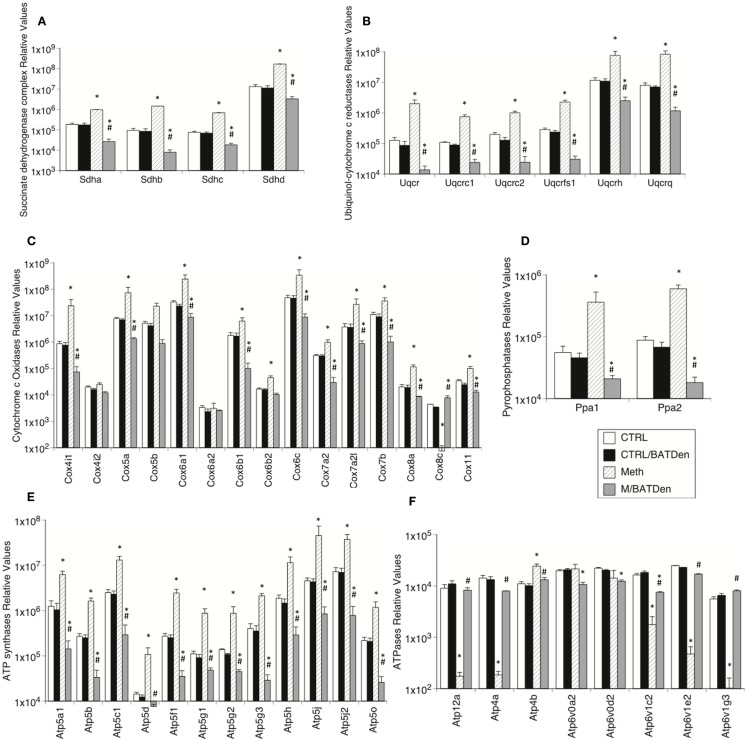
**Levels of mitochondrial electron-transport molecules in BAT from animals injected with Methamphetamine**. Transcriptional enzymes were measured by SyBrGreen qRT-PCR. Relative values were normalized based on GAPDH. Levels of **(A)** Complex II succinate dehydrogenases, **(B)** Complex III ubiquinol-cytochrome *c* reductases, **(C)** Complex IV cytochrome *c* oxidases, **(D)** pyrophosphatases, **(E)** ATP synthases, and **(F)** ATPases. Values represent the average ± SEM of six animals/group. Values represent the average ± SEM of six animals/group. *p < 0.05 Compared to respective controls, ^#^*p* < 0.05 compared to Meth group.

Other mitochondrial complexes also followed a similar pattern, with the transcription of their genes being upregulated by Meth, but downregulated in Meth-injected BAT-denervated animals (Figure 5). This was the case for Complex II succinate dehydrogenases (Figure 5A, ANOVA *p* < 0.001), Complex III ubiquinol-cytochrome *c* reductases (Figure 5B, ANOVA *p* < 0.001), Complex IV cytochrome *c* oxidases (Figure 5C, *p* < 0.001), pyrophosphatases (Figure 5D, *p* < 0.001), and ATP synthases (Figure 5E). However, most ATPases followed an inverse pattern, with Atp12a, 4a, 1c2, 1e2, and 1g3 being downregulated by Meth, and upregulated by denervation. Atp4b, 6v0a2, and 6v0d2 were unchanged by Meth (Figure 5F, *p* < 0.01).

**Figure 6:**
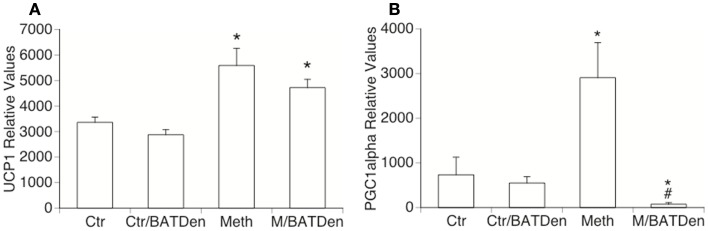
**Thermogenesis-related genes in BAT from Meth-injected animals**. **(A)** UCP1 and **(B)** PGC1alpha transcriptional levels were measured by SyBrGreen qRT-PCR. Relative values were normalized based on GAPDH. Values represent the average ± SEM of six animals/group. *p < 0.05 Compared to respective controls, ^#^*p* < 0.05 compared to Meth alone.

The expression of UCP1 transcripts was increased by Meth both in the BAT of sham-operated mice, and in denervated animals. This suggests that UCP1 upregulation by Meth is not sympathetically mediated (Figure 6A). This also suggests that sympathetic mediators act by controlling mitochondrial respiratory chain complexes, while induction of UCP1 and ATPases may be alternatively regulated, either directly or by sympathetically independent pathways activated upon Meth use. We have determined that BAT participates on the development of Meth hyperthermia in correlation with the SNS-mediated activation of mitochondrial respiratory chain complexes. Conversely, the expression of PGC1alpha was significantly enhanced by Meth, and prevented by denervation (Figure 6B).

**Figure 7:**
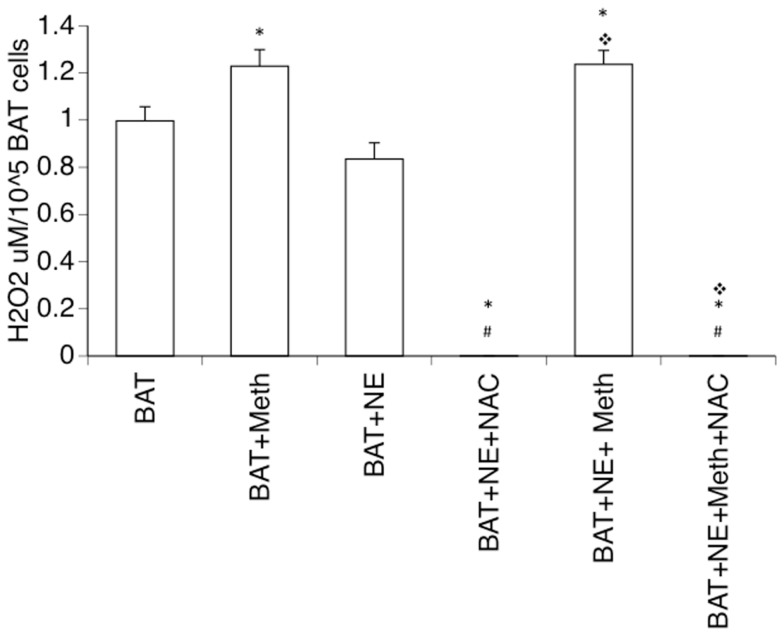
**Production of hydrogen peroxide by Meth-stimulated mature brown adipocytes**. Single cell suspensions of BAT were obtained from adult mice and placed in culture, and were immediately stimulated with 60 μM of Meth, 1 ng/ml of Norepinephrine (NE), 5 μM of *N*-acetyl-d-cysteine (NAC), or combinations of these reagents. Hydrogen peroxide was detected in the cultures for 60 min. Results correspond to the average of four independent readings. *p < 0.05 Compared to respective controls, ^#^*p* < 0.05 compared to Meth alone.

One important consequence of Meth, described in cell types such as neurons and astrocytes, is the induction of ROS, which is due to the enhancement of mitochondrial respiratory chain activation (Lau et al., 2000; Wu et al., 2007). We checked whether Meth induces ROS production in isolated brown adipocytes either by direct or by sympathomimetic stimulation. We found that Meth was directly capable of inducing H_2_O_2_ production in brown adipocytes in culture (Figure 7), whereas NE neither elicited a ROS production response in BAT cells nor affected the increase of ROS caused by Meth. The addition of NAC, a glutathione precursor, completely abolished the production of H_2_O_2_.

**Figure 8:**
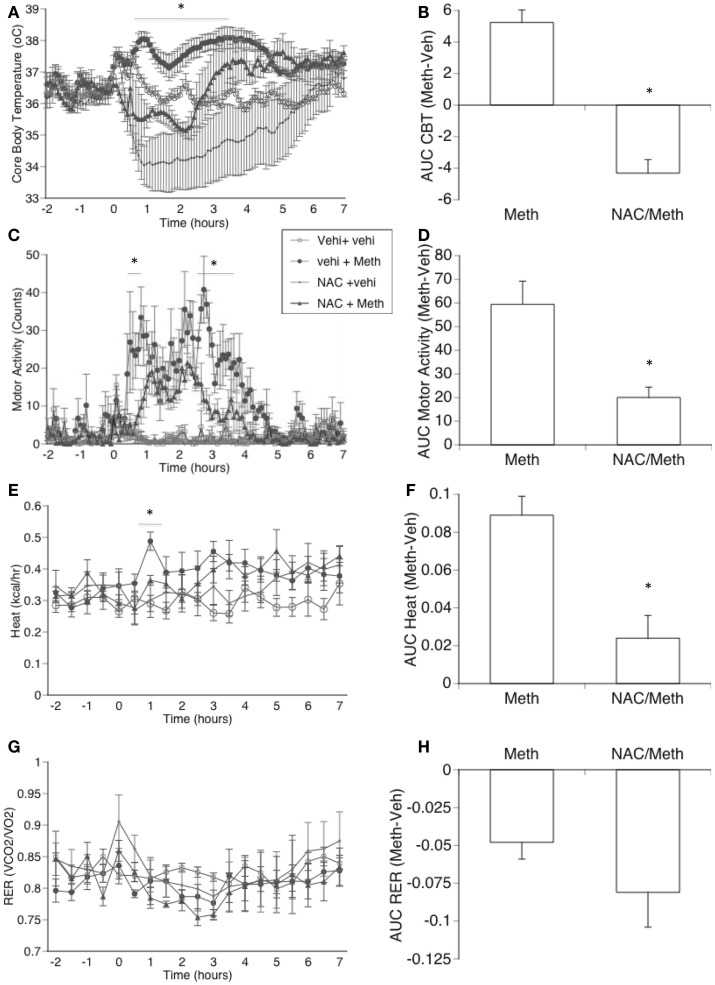
**Effect of *in vivo* ROS blockage in Meth hyperthermia and metabolic parameters**. Mice implanted with telemetry devices were IP-treated with NAC (1000 mg/kg) or vehicle 60 min before the injection of Vehicle or Meth (3 mg/kg), and were evaluated during 6 h for core body temperature **(A)**, motor activity **(C)**, Heat production **(E)**, and RER **(G)**. Stress peak (first hour) was excluded. Values represent the average ± SEM of four animals/group. *p < 0.05 compared to NAC control, *n* = 4, Student *t*-test. Area under the curve (AUC) was used to quantify changes in **(B)** CBT, **(D)** locomotor activity, **(F)** heat, and **(H)** RER in Meth compared to vehicle-injected animals, and to investigate the effect of NAC. Values represent the average difference between Meth-injected and Vehicle-injected respective controls ± SEM, for four animals/group. *p < 0.05, ANOVA followed by Bonferroni’s *post hoc* test, *n* = 4 animals/group.

It has been suggested that ROS plays a modulatory role in metabolism and thermogenesis (Bell et al., 2006). Furthermore, ROS production has been linked to mitochondrial functions, in addition to activating uncoupling proteins (Pecqueur et al., 2001; Rial and Zardoya, 2009). Thus, we investigated whether ROS has a role in the induction of Meth hyperthermia. We performed a blockage of ROS *in vivo* through the intraperitoneal administration of NAC while recording CBT, MA, and metabolic parameter. We found that the decrease in ROS availability caused hypothermia in control animals (Figure 8A). In addition, this ROS limitation significantly delayed the increase in temperature in Meth-injected mice, where temperature did not reach levels above 37.2°C. The calculation of the AUC in Meth-injected animals normalized to the respective control groups showed that ROS blockage significantly impacted the development of hyperthermia (Figure 8B). This result suggests a role for ROS in Meth hyperthermia and in the overall control of CBT. ROS blockage *in vivo* also transiently affected MA (Figures 8C,D), replicating the effects of BAT denervation. ROS blockage affected heat production in a transient (Figure 8E) but significant way (Figure 8F). RER was not affected by ROS blockage (Figures 8G,H).

Reactive Oxygen Species production is largely modulated by Protein Kinase C delta (PKCδ) (Kohda and Gemba, 2005). However, Meth injection in PKCδ KO animals did not affect CBT, heat, MA, or RER, suggesting that ROS produced in response to Meth in BAT, and in other sites, may follow a PKCδ – independent pathway (not shown).

## Discussion

The role of BAT thermogenesis in Meth-induced hyperthermia has been previously overlooked. Here, we have identified BAT as a major site of thermogenesis upon activation by Meth, contributing to the elevation of CBT after a single dose of Meth, and potentially modulating MA. Sympathetically mediated mitochondrial respiratory chain complex activation seems to be fundamental to thermogenesis induced by Meth in BAT, even though UCP1 and ATPases are induced by Meth regardless of intact nerve input. However, PKCdelta regulated processes, such as mitochondrial oxidative stress (Kohda and Gemba, 2005), which is highly active in BAT under sympathetic control (Prusiner et al., 1968a,b), may not have an important influence in temperature in Meth-injected animals, since KO animals showed the same hyperthermic profile as control animals (not shown).

Both ablation and denervation of interscapular BAT, the largest BAT deposit, decreased the length of hyperthermia, revealing an important influence of this site on temperature. Denervation (Rothwell and Stock, 1984) prevented the upregulation of mitochondrial electron transport chain genes induced by Meth, and decreased temperature. It is possible that this effect on temperature is underrepresented, due to residual BAT deposits existing in other parts of the body. But the significant effect of denervation on electron transport chain molecule transcripts suggests that mitochondrial metabolism, which is largely responsible for BAT thermogenesis, may be triggered by Meth-induced epinephrine (Kuczenski et al., 1995) that reaches the tissue through sympathetic nerves (Bartness et al., 2010). Furthermore, the analysis of the AUC revealed that BAT denervation significantly decreased MA induced by Meth. The effect of ablation on MA was not as strong. However, when compared to sham animals stimulated with Meth, the MA profile of BAT ablated and denervated animals showed that both ablation and denervation in fact decreased the length of motor activation. Interestingly, the limitation of ROS availability by the administration of NAC *in vivo* also affected MA. The connection between these findings remains to be investigated.

There were important differences between the ablation and denervation of BAT in regards to RER (VCO_2_/VO_2_) and heat production. Denervation, in particular, significantly affected both. In vehicle-injected control animals, both ablation and denervation transiently decreased RER following injection. In Meth-injected mice, where RER decreased in all groups, BAT ablation accelerated the recovery to baseline, whereas BAT denervation, but not ablation, prevented the increase of heat production induced by Meth. It is also important to keep in mind that heat as a metabolic parameter does not necessarily depend on, or correlate with CBT. The decrease in heat in BAT denervation is related to a lower VO_2_ consumption, which could be a result of decreased number of mitochondria, and to other metabolic changes in BAT. In contrast, the RER ratio decreased in Meth-injected mice, regardless of BAT presence or enervation, suggesting that acute Meth may transiently change the metabolic balance toward lipolysis in a sympathetically independent fashion. However, in denervated animals the RER ratio showed a faster recovery. This has been shown in animals subcutaneously injected with Meth, and the fact that propanolol corrects the RER ratio suggests that white adipose tissue (WAT) in the muscle may have a thermogenic role (Estler et al., 1970). Thus, it is possible that in BAT-denervated animals, WAT acquires a thermogenic role as well. And while the mechanisms are not clear, it is possible that there is a common molecular basis for the thermogenic actions of BAT and of WAT within the muscle. Interestingly, the suppression of interscapular BAT, either by ablation or denervation, decreased MA of skeletal muscles. In fact, denervation of BAT in Meth-injected animals drastically decreased motor activation and reduced heat dissipation, suggesting that molecular changes in BAT deprived of b-adrenergic input affect other sites, including muscle and skin (Nakamura and Morrison, 2007). Modifications caused in BAT by denervation in combination with potential alternative actions of Meth or the decrease in mitochondria activity, could account for actions in other sites. However, further studies may be needed clarify this question.

The impact of Meth on mitochondrial function and the SNS regulation in BAT are detectable upon analysis of mitochondrial morphology. Meth does not change the size or number of mitochondria in BAT, suggesting that at a single dose it does not affect mitochondria biogenesis or autophagy. However, it does decrease cristae density, changing of the surface area for electron transport and ATP synthesis, which may also validate the lower respiratory rate seen in BAT stimulated with Meth (Guderley et al., 2005). The number and size of mitochondria, as well as cristae morphology, were highly modulated by sympathetic input. In denervated BAT groups, regardless of Meth, mitochondria were smaller, occurred in significantly smaller numbers and had significantly denser cristae. However, in denervated animals, Meth further decreased the number of mitochondria. Changes in both mitochondrial morphology and the levels of electron transport chain molecules are generally correlated with ROS induction. Puzzling enough, NE, a sympathetic neurotransmitter present in BAT and upregulated by Meth (Sprague et al., 2003, 2007), was not able to increase H_2_O_2_ production to above baseline levels on isolated mature BAT cells. Meth, in contrast, directly induced a significant increase of H_2_O_2_. Thus, the further decrease in mitochondria size and number in Meth-injected BAT-denervated animals could be a result of damaging effects of ROS locally induced by Meth, and could show a potential role for sympathetic neurotransmitters in preserving mitochondrial function.

Blockage of ROS availability *in vivo*, with the administration of a glutathione precursor, not only prevented hyperthermia, but also induced a transient hypothermia. Thus ROS may be important to signal changes in CBT. However, we cannot exclude the possibility that in *in vivo* blockage, ROS production by other thermogenic sites, such as muscle, might be transiently compromised. Despite this, we have estimated a 40% contribution of BAT to the hyperthermia induced by Meth. The data suggests that while mitochondria morphology and the amount of electron transport chain molecules in BAT might be under control of sympathetic input, ROS, which seemed to play a major role in Meth hyperthermia, might rather be directly induced by the drug. Meth is also able to directly induce ROS in other cell systems such as macrophages (data not shown), suggesting that Meth may act as a redox cycler compound. Previous studies in different models have suggested that ROS have toxic effects that are enhanced by hyperthermia (Lin et al., 1991), and that ROS also work as signaling components for apoptosis induced by hyperthermia (Katschinski et al., 2000). To our knowledge, this is the first report of the involvement of ROS in the development of the hyperthermia phenomenon.

The blockage of ROS *in vivo* correlated with a decrease in MA, suggesting that the action of Meth in other thermogenic sites such as muscle may contribute to Meth hyperthermia. Blockage of ROS *in vivo* also affected heat production, as a result of decreased oxygen consumption, which may result from the initial hypothermia induced in animals treated with the glutathione precursor NAC. As a mitochondria-rich site, BAT may be crucial.

In contrast to the energy-storing white fat, BAT is an important component in peripheral CBT control, as it is a thermogenic site of energy dissipation. Under basal conditions, respiration is coupled to phosphorylation in brown fat cells, and epinephrine stimulates a direct uncoupling action (Reed and Fain, 1968a,b) to produce heat (Prusiner et al., 1968a,b; Reed and Fain, 1968a,b). When activated by epinephrine, β3-adrenoceptors (β3-AR) in BAT mediate thermogenesis and induce lipolysis in WAT (Preitner et al., 1998; Collins and Surwit, 2001; Collins et al., 2001; Arch, 2002; Inokuma et al., 2005, 2006). In BAT, heat is generated by the activation of UCP1, which is present in the inner mitochondrial membrane of brown adipocytes (Nedergaard et al., 2001a,b; Erlanson-Albertsson, 2003; Sell et al., 2004). UCP1 is a regulated proton carrier that short circuits the proton gradient generated by the respiratory chain, thereby uncoupling ATP synthesis from respiration and releasing heat from oxidation of substrates (Arechaga et al., 2001; Ledesma et al., 2002). Although BAT UCP1 does not contribute to basal energy (Matthias et al., 1999, 2000), it is the site of adaptative thermogenesis, providing extra heat in hibernating animals, newborns, and cold-exposed mammals (cold-induced thermogenesis), as well as maintaining energy balance in response to diet (diet-induced thermogenesis) (Bachman et al., 2002; Feldmann et al., 2009).

Meth also significantly affected the mitochondrial electron transport chain in BAT, as detectable by an elevation in the transcriptional levels of redox carriers. BAT denervation not only prevented mitochondrial activation, but also suppressed the expression of electron transport chain genes to below baseline levels. Another adaptative response of BAT to the injection of Meth was an increase in the expression of UCP1. However, even though UCP1 in BAT has been described to be under control of sympathetic nerves, its levels were not significantly altered by denervation after Meth injection. Conversely, ATPases were mostly down regulated by Meth, and brought back to normal levels by denervation. In contrast, the expression of PGC1alpha, a co-activator of UCP1 transcription that is highly upregulated by Meth, was completely abolished by denervation in Meth-injected mice. This pathway suggests a role for SNS in promoting thermogenesis, and suggests the participation of UCP1 and PGC1a in addition to ROS, in Meth hyperthermia.

We have demonstrated that BAT plays an important role in Meth thermogenesis, and that the SNS mediates the activation of mitochondrial electron transport chain molecules and PGC1a in BAT, by modulating their transcriptional levels. We have shown that ROS is directly induced in BAT by Meth, and that this can be through a SNS-independent path. We have demonstrated that ROS play an important role in generating hyperthermia *in vivo*. Meth provides signals that trigger mechanisms of adaptative thermogenesis. NE induced by Meth (Wagner et al., 1979) may regulate mitochondrial activation and UCP1, while direct induction of ROS by Meth may signal temperature elevation mechanisms and affect mitochondrial performance. Targeting of these pathways may provide insights into a way to rescue drug abusers in hyperthermic conditions.

## Conflict of Interest Statement

The authors declare that the research was conducted in the absence of any commercial or financial relationships that could be construed as a potential conflict of interest.
